# Combination effect of therapies targeting the PI3K- and AR-signaling pathways in prostate cancer

**DOI:** 10.18632/oncotarget.12771

**Published:** 2016-10-20

**Authors:** Shalini Singh Yadav, Jinyi Li, Jennifer A. Stockert, James O'Connor, Bryan Herzog, Cordelia Elaiho, Matthew D. Galsky, Ashutosh Kumar Tewari, Kamlesh Kumar Yadav

**Affiliations:** ^1^ Department of Urology, Icahn School of Medicine at Mount Sinai, New York, NY 10029, USA; ^2^ Division of Hematology and Medical Oncology, Icahn School of Medicine at Mount Sinai, New York, NY 10029, USA

**Keywords:** prostate cancer, drug-resistance, drug combination, synergy, antagonism

## Abstract

Several promising targeted-therapeutics for prostate cancer (PCa), primarily affecting the androgen receptor (AR) and the PI3K/AKT/mTOR-pathway, are in various phases of development. However, despite promise, single-agent inhibitors targeting the two pathways have not shown long-term benefits, perhaps due to a complex compensatory cross talk that exists between the two pathways. Combination therapy has thus been proposed to maximize benefit. We have carried out a systematic study of two-drug combination effect of MDV3100 (AR antagonist), BKM120 (PI3K inhibitor), TKI258 (pan RTK inhibitor) and RAD001 (mTOR inhibitor) using various PCa cell lines. We observed strong synergy when AR-positive cells are treated with MDV3100 in combination with any one of the PI3K-pathway inhibitors: TKI258, BKM120, or RAD001. Growth curve based synergy determination combined with Western blot analysis suggested MDV3100+BKM120 to be the most effective in inducing cell death in such conditions. In the case of dual targeting of the PI3K-pathway BKM120+TKI258 combination displayed exquisite sensitivity in all the 5 cell lines tested irrespective of androgen sensitivity, (LNCaP, VCaP, 22Rv1, PC3 and Du145). The effect of blockade with BKM120+TKI258 in PC3 cells was similar to a combination of BKM120 with chemotherapy drug cabazitaxel.

Taken together, our observation supports earlier observations that a combination of AR-inhibitor and PI3K-inhibitor is highly synergistic. Furthermore, combining BKM120 with TKI258 has better synergy than BKM120+RAD001 or RAD001+TKI258 in all the lines, irrespective of androgen sensitivity. Finally, BKM120 also displayed synergy when combined with chemotherapy drug cabazitaxel. No antagonism however was observed with any of the drug combinations.

## INTRODUCTION

Prostate cancer (PCa) is the most common cancer in American men and the second-leading cause of the approximately 28,000 cancer-related deaths each year [1]. Although initially responsive to androgen deprivation therapy (ADT), nearly all metastatic PCa progress to a castrate-resistant prostate cancer (CRPC) phase with poor prognosis [2]. Although some CRPCs respond to chemotherapeutic drugs, like docetaxel [3] or cabazitaxel [4], the benefit is limited and often short-lived. Recent studies have cemented earlier observations that deregulation of androgen-receptor (AR) signaling and the PI3K/AKT/mTOR pathways play important roles in carcinogenesis, progression and development of resistance [5–7].

The AR-signaling pathway has been the main therapeutic target for more than 70 years and ADT shows remarkable beneficial effect in controlling the early phase of the disease. Studies directed towards understanding the mechanism of CRPC development identified upregulation of AR-signaling in more than 60% of CRPCs, either through overexpression, mutation or AR splice-variant production [8]. This prompted the development of new AR-signaling blockers Enzalutamide and Abiraterone, both of which recently acquired FDA approval [9–12]. Since Enzalutamide directly binds and inhibits AR function it has also been approved for treatment-naïve patients [11].

The PI3K-AKT-mTOR pathway on the other hand is important for normal growth and survival of the cell. It has been shown to be upregulated in 30-50% of all prostate cancers [13]. PTEN alone is lost in more than 40% of highly lethal CRPCs [14] and around 15% of primary prostate cancer [7]. Thus, small molecule inhibitors targeting critical members of this pathway are in various stages of development or clinical trials, including BKM120 [15], TKI258 [16] and RAD001 [17]. Whereas BKM120 (a PI3K inhibitor) and RAD001 (an mTOR inhibitor) directly inhibit key members of the PI3K-pathway, TKI258 is a pan-receptor tyrosine kinase inhibitor (targeting FGFR, VEGFR, PDGFR, etc) that has been shown in breast and colorectal cancer models to act primarily through inhibition of the PI3K-pathway signaling [18, 19].

Despite the availability of the new targeted-chemotherapeutics, treatment of patients is impeded by the emergence of drug resistance. For instance, CRPC patients on enzalutamide therapy develop resistance within 2 years [20] and single agent RAD001 and BKM120 treatment have failed clinical trials [21, 22]. Remarkably, a crosstalk between the AR- and PI3K-pathway has been established where the loss of AR leads to upregulation of the PI3K-signaling pathway, suggesting a need for simultaneous targeting of both the pathways [23, 24]. Indeed, combination therapy regimens in breast cancer, another hormone-dependent system, have shown not only to improve therapeutic efficacy and reduce drug toxicity but also delay drug resistance development [25]. In PCa combined inhibition of AKT (with AZD5363) and AR (with MDV3100 or bicalutamide) has been shown to be effective in delaying PCa progression in preclinical models [26, 27]. However, with several different drugs targeting different members of the PI3K-signaling cascade, a robust and facile method of determining the best synergistic combination is required. Moreover, determining the nature of interaction of two drugs to be combined is essential in making therapeutic choices for the most favorable outcome. Some drug combinations can be antagonistic and would require more of each drug to achieve similar outcomes [28]. Or, they can synergize and increase each others' effect thus requiring lower overall dose and lesser toxicity [29]. Finally, identifying the critical targeting node in a signaling pathway will enable designing better combination for complete pathway inactivation. In the era of precision medicine, identifying the complex nature of drug interaction will be helpful in formulating treatment regimens that are appropriate and likely to prolong remission.

**Figure 1:**
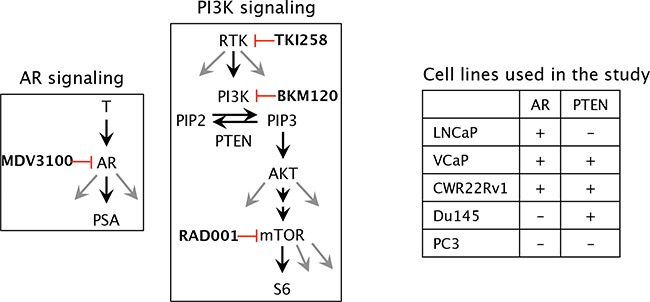
Schematic of the molecular targets of the drugs and the cell lines chosen in the study T, testosterone; AR, androgen receptor; PSA, prostate specific antigen; MDV3100 (enzalutamide), (RTK, receptor tyrosine kinase; PI3K, phosphoinositide-3 kinase; PIP2, Phosphatidylinositol (4,5)-bisphosphate; PIP3, Phosphatidylinositol (3,4,5)-trisphosphate; PTEN, Phosphatase and tensin homolog /phosphatidylinositol-3,4,5-trisphosphate 3-phosphatase; AKT, a serine/threonine protein kinase, mTOR, mammalian target of rapamycin; TKI258 (Dovitinib, pan RTK inhibitor); BKM120 (buparlisib, PI3K inhibitor); RAD001 (everolimus, mTOR inhibitor).

In this study we used an AR antagonist and several PI3K-pathway inhibitors (Figure 1) targeting various nodes of the complex signaling pathway and tested their as single agents or in combination in several prostate cancer cell lines. The WST-1 assay in combination with the CompuSyn algorithm and western blot analyses were utilized to compute and validate the combination indices (CI) of combined-treatment effect of Enzalutamide (MDV3100, AR antagonist), Dovitinib (TKI-258, pan RTK inhibitor), Buparlisib (BKM120, pan PI3K inhibitor), Everolimus (RAD001, mTOR inhibitor) and chemotherapy drug Cabazitaxel in different PCa cell lines. Not only does our data confirm previous observations of combining AR- and PI3K-pathway inhibition for better efficacy, it identifies the targeting node on the PI3K axis for maximal inhibitory effect. Our study also identified that dual targeting of upstream targets (such as receptor tyrosine kinases) with an AKT level blockade (with PI3K inhibitor) is most synergistic.

## RESULTS

### Single-agent drug treatment

We began by testing the sensitivity of the PCa cell lines to single-agent treatments. For this, we generated the dose-response curves and calculated the IC_50_ values of single-agent inhibitors in LNCaP (Supplementary Figure S2), PC3 (Supplementary Figure S3) and 22Rv1 (Supplementary Figure S4) and Table 1. In LNCaP cells, the IC_50_ value determined for MDV3100 was relatively high at around 6.31 μM similar to what has previously been reported [23, 30]. However, the IC_50_ values for TKI258, BKM120 and RAD001 were 3.4μM, 3.23μM and 4.05μM respectively. In the case of PC3 cells, IC_50_ values for TKI258, BKM120 and RAD001 were determined to be 2.57μM, 2.81μM and, 5.46μM respectively. The increased IC_50_ in the case of RAD001 is perhaps due to the inactivation of PTEN in both LNCaP and PC3 cells (via either frameshift mutation or homozygous deletion, respectively), which drives constitutive PI3K-pathway activation. Indeed, 22Rv1 cells, which express wild-type PTEN, have increased sensitivity to RAD001 at 1.57μM and BKM120 at 1.7μM (Supplementary Table S1 and Supplementary Figure S4), similar to that reported with CH5132799, another mTOR inhibitor in the same cell line [31].

**Table 1:** IC50 of single inhibitor treatment (μM)

	MDV3100	TKI258	BKM120	RAD001	Cabazitaxel
LNCaP	6.31±2.03	3.04±1.20	3.23±1.32	4.05±1.51	1.87±1.17
PC3	nd	2.57±1.26	2.81±1.15	5.46±1.41	6.31±1.34
22Rv1	237.4±3.8	4.38±1.19	1.7±1.23	1.57±1.44	nd

nd = not determined

**Figure 2:**
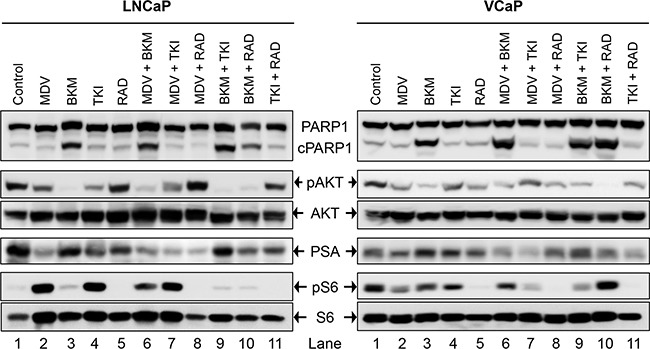
Biochemical effect of perturbations with various drugs, affecting the AR- and PI3K-signaling pathways, and their two-drug combinations in two PCa lines; LNCaP (left), and VCaP (right) Acute signaling blockage was generated by treating cells with 2xIC_50_ of the mentioned drug(s) for 1 day (MDV3100 10μM, BKM120 6 μM, TKI258 6 μM, RAD001 10 μM). The cell lysate of the treated cells was subjected to western blot analysis using the indicated antibodies.

To test whether each of the drugs was able to physiologically inhibit its intended target, western blot analysis was performed on whole-cell lysates from PCa cells treated acutely for 1 day with a high concentration (2xIC_50_) of the drugs. This treatment was done to exclude the physiological effect of pathway inhibition (cell death, altered growth profile, etc) from the direct effect of signaling block. LNCaP cells treated with MDV3100 displayed loss of PSA expression, Figure 2 (lane 2). Whereas BKM120 treatment resulted in almost complete loss of AKT phosphorylation, the effect was partial with TKI258 treatment, Figure 2 (lanes 3 and 4). Treatment with RAD001 led to the complete loss of S6 phosphorylation without affecting AKT phosphorylation, Figure 2 (lane 5). Similar results were observed in other PCa cell lines; VCaP, (Figure 2), PC3, 22Rv1 and Du145 (Figure 4). These observations are consistent with expected response of downstream effectors upon the inhibition of upstream targets, Figure 1.

### Combination treatment

To identify whether a drug combination was synergistic, the combination index (CI) of each drug-pair was calculated. For this, cells were treated with seven inhibitor combination ratios, taking three concentrations above, and three below the approximate IC_50_ value of each drug as described in the Methods section. Thereafter, using survival data generated from the WST-1 assays, CI-Fa, isobologram and dose-response plots were generated for each drug-pair. To identify whether a drug combination had a synergistic or antagonistic effect, each above-mentioned factor was integrated using the CompuSyn algorithm and CI values were calculated, both at Fa 0.5 (50% cell death) and Fa 0.8 (80% cell death), Table 2. CI values less than 1 denotes synergism and greater than 1 antagonism. Finally, the fold changes affected due to the drug-combinations were also tabulated, Supplementary Table S1.

**Table 2:** CI values of combination treatment (Fa=0.5/0.8)

	MDV-TKI	MDV-BKM	MDV-RAD	TKI-BKM	TKI-RAD	BKM-RAD	Cab-BKM	Cab-TKI	Cab-RAD
LNCaP	0.33/0.74	0.26/0.37	0.11/0.19	0.20/0.28	0.19/0.28	0.34/0.35	0.46/0.49	0.58/0.93	0.74/0.77
PC3				0.77/1.14	0.65/0.68	0.54/0.77	0.47/0.83	0.59/0.63	0.72/0.88
22Rv1				0.73/0.67	0.4/0.88	0.41/0.36			

Synergy <1, Additive 1, Antagonistic >1

MDV3100 combinations for PC3 and 22Rv1, and Cabazitaxel combinations for 22Rv1, were not determined

### Targeting both AR and PI3K pathways simultaneously

**Figure 3:**
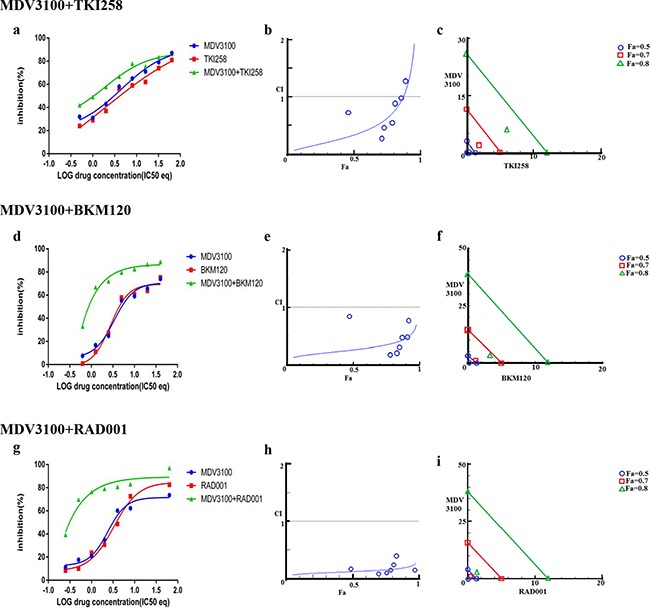
Combination effect of therapies targeting the AR- and PI3K pathway LNCaP cells were treated with the indicated combination of the drugs and analyzed as described in Methods and Supplementary Figure S1. Left column (**a, d** and **g**): Curve shift analysis. Degree of left shifted indicated the amount of synergism with the indicated drug-combination. Middle column (**b, e** and **h**): Fa-CI plot. Blue values above the gray horizontal ‘additive’ line indicates trends towards antagonism. Low CI values with increased in Fa values suggest better compatibility and high synergism between the drugs. Right column (**c, f** and **i**): Isobolograms: The green, red and blue lines indicate where the theoretical additive line is for a particular Fa value (here, green, Fa 0.8, red Fa 0.7 and blue Fa 0.5. The closer the calculated values fall towards the origin, the greater the synergy between the drugs.

**Figure 4:**
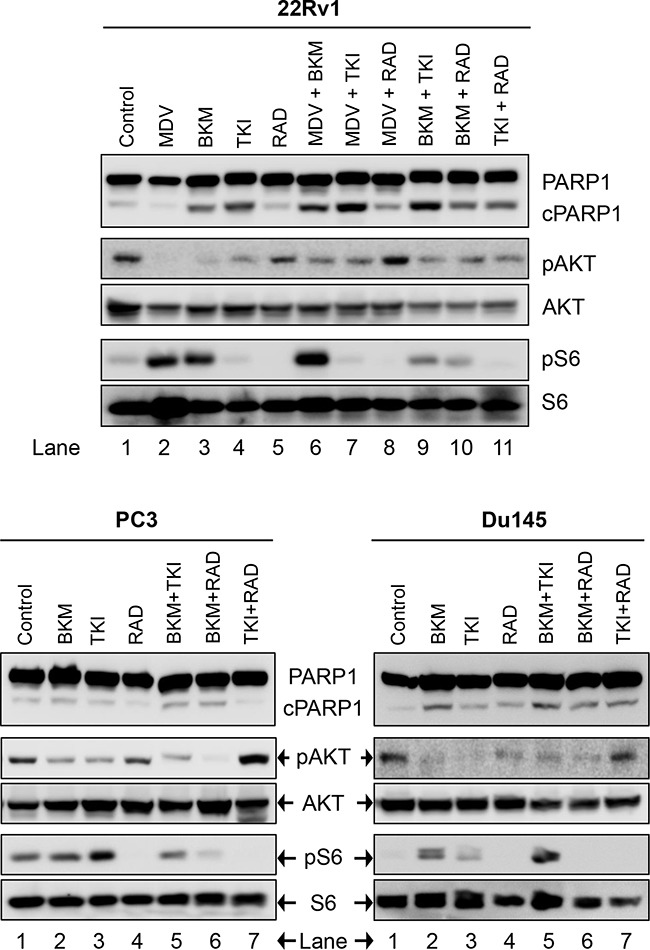
Biochemical effect of perturbations with various two-drug combinations in three CRPC PCa lines: 22Rv1, PC3, and Du145 Cells were treated and analyzed in Figure 2.

The dependency of PCa on AR- and PI3K-pathways provides an ideal therapeutic opportunity for a combination treatment that inhibits both the pathways simultaneously. Indeed, when LNCaP cells were treated with AR antagonist MDV3100 in combination with a PI3K pathway inhibitor (TKI258, BKM120 or RAD001) various levels of synergy were observed. The strongest synergism was observed for MDV3100+RAD001 (CI 0.11), followed by MDV3100+BKM120 (CI 0.26), and finally MDV3100+TKI258 (CI 0.33). Remarkably, in the case of MDV3100+RAD001 treatment, synergy was observed even at highest concentration with Fa 0.7 and Fa 0.8 points in the isobologram segregated at the origin, Figure 3i. Moreover, the CI-Fa plot showed all points to be close to the horizontal axis at all concentrations, Figure 3h. This was in contrast to MDV3100+TKI258 combination where at high drug concentration, the curve tended towards additive (CI =1), Figure 3b and Table 2. Furthermore, whereas in the case of MDV3100+RAD001 this translated to a 90 and 19 fold reduction in drug dose for MDV3100 and RAD001 respectively to achieve 50% inhibition as compared to single-agent treatment, in the case of MDV3100+TKI258 or MDV3100+BKM120, the synergistic effects were equivalent to a reduction of drug dose by 42 and 7, and 57 and 7 folds respectively, Table 3. Analysis of the dose-response curves generated upon combining MDV3100 with TKI258, BKM120 or RAD001 in LNCaP cells (Figure 3, first column) suggested the best shift to be generated upon BKM120 or RAD001 combination, Figure 3g. To determine the best combination between MDV3100+BKM120 and MDV3100+RAD001, we carried out western blot analysis upon 1-day treatment with various drug-combinations at 2x IC_50_ concentrations. The drug concentration was chosen to make comparison consistent with single agent treatment. The level of downstream effector inhibition and cleaved poly ADP ribose polymerase 1 (cPARP1), shown to be a robust read out for apoptosis [32], upon treatment was evaluated. Remarkably, contrary to the CI values, the level of cPARP1 was much higher in cells treated with MDV3100+BKM120 compared to cells treated with MDV3100+RAD001, Figure 2 (left, lane 6, 7 and 8). Grown in charcoal stripped media (to mimic castrate conditions) also showed similar results, Supplementary Figure S5. This could be a ramification of acute treatment of the cells, or perhaps MDV3100+RAD001 induces more of a cytostatic effect whereas MDV3100+BKM120 induces apoptosis. Further experiments are required to differentiate between these two effects. The same effect was observed in the AR positive cell line VCaP when treated similarly Figure 2 (right, lanes 6,7 and 8). We further validated this observation in another AR-positive but CRPC cell line 22Rv1, which expresses a combination of full-length and a splice variant form of AR that lacks the ligand-binding domain making it insensitive to anti-androgens [33]. Similar to observations in LNCaP and VCaP cells, MDV3100+BKM induced higher levels of cPARP1 in 22Rv1 cells when compared to MDV3100 or BKM120 alone, Figure 4 (lane 6). However, we observed a slightly higher level of cell death induced with the MDV3100+TKI258 combination, Figure 4 (lane 7). Perhaps the cells are still wired, in part, for survival through the full-length AR signaling pathway and signaling upstream of PI3K. Taken together, this indicates that combining MDV3100 with BKM120 is highly synergistic. This finding is in line with earlier observations where a combination of MDV3100 with CH5132799 (an AKT inhibitor) was shown to be effective in delaying resistance development in preclinical models of PCa [26, 27].

**Table 3:** IC50 value (μM) of combination treatment and drug-dose reduction (Fa=0.5)

		MDV3100-TKI258		MDV3100-BKM120		MDV3100-RAD001		TKI258-BKM120		TKI258-RAD001		BKM120-RAD001		Cabazitaxel –BKM120		Cabazitaxel –TKI258		Cabazitaxel –RAD001	
LNCaP	Combination IC50	0.15	0.46	0.11	0.35	0.07	0.21	0.32	0.32	0.33	0.33	0.60	0.60	0.89	0.45	0.35	0.70	0.64	1.29
	Fold reduction	42.07	6.61	57.36	9.23	90.14	19.29	9.50	10.09	9.21	12.27	5.38	6.75	2.02	7.18	5.14	4.34	2.81	3.14
PC3	Combination IC50							1.24	0.82	1.73	0.86	1.64	0.65	1.41	0.71	1.31	0.99	2.01	2.01
	Fold reduction							2.08	3.44	1.49	6.35	1.72	8.40	4.48	3.97	4.82	2.61	3.14	2.72
22Rv1	Combination IC50							0.09	0.15	0.57	0.2	0.01	0.04						
	Fold reduction							10.78	1.53	4.96	5.1	6.00	5.75						

MDV3100 combinations for PC3 and 22Rv1, and Cabazitaxel combinations for 22Rv1, were not determined

**Figure 5:**
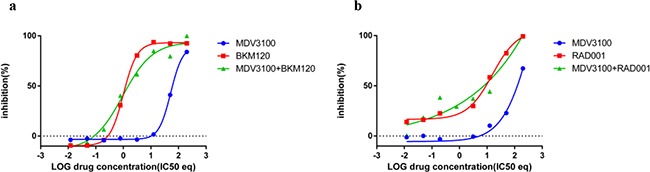
Combination effect of PI3K inhibitors with MDV3100 in the AR negative cell line PC3 PC3 cells were treated with **a.** MDV3100+BKM120 combination **b.** MDV3100+RAD001 and analyzed as described in Methods and Figure 3.

### Combined targeting of PI3K pathway

**Figure 6:**
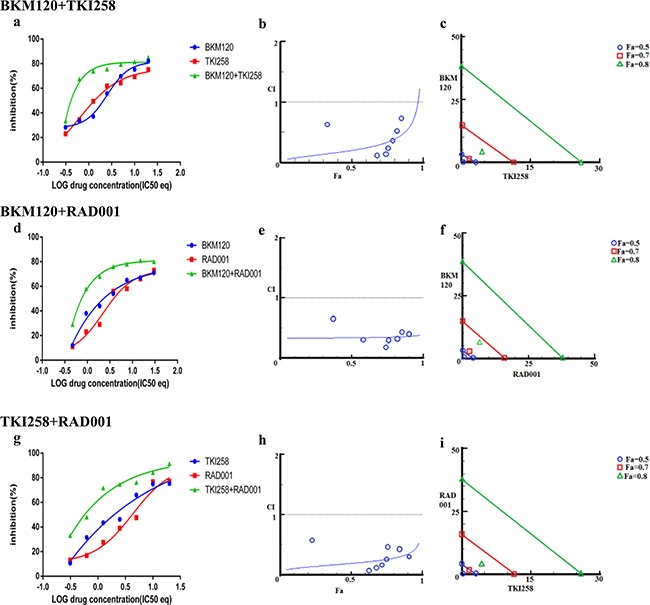
Combination effect of two drugs that target the PI3K-pathway simultaneously: LNCaP cells were treated with the indicated combination of the drugs and analyzed as described in Methods and Figure 3

More than 49% of the somatic alteration in metastatic CRPC [6] and around 23% in primary PCa have been associated directly with the PI3K pathway [7] underscoring the dependence of PCa on this pathway. Since the pathway is multi-nodal, identifying the correct combination is crucial for effective abrogation of the pathway in relation to PCa. To achieve this we combined BKM120+TKI258, BKM120+RAD001 and TKI258+RAD001 and tested their effect on various PCa cell lines. When LNCaP cells were treated with combinations of BKM120+TKI258, BKM120+RAD001 or TKI258+RAD001, we observed CI values of 0.20. 0.19 and 0.34, respectively, Table 2. Remarkably, the CI at Fa 0.8 remained similar to Fa 0.5 in this cell line for all the combinations, Table 2 and Figure 6 middle column, suggesting the drugs to be well tolerated for high drug combinations. However, between the three, the least amount of synergism was observed for the TKI258+RAD001 combination, which was confirmed with western blot analysis where the levels of cPARP were least, when compared to BKM120+TKI258 BKM120+RAD001 levels, Figure 2 (lane 11). This was also observed when VCaP cells were treated similarly. However, whereas in VCaP cells both BKM120+TKI258 and BKM120+RAD001 combinations led to increased cPARP1 levels, it was only observed with the BKM120+TKI258 combination in LNCaP cells, Figure 2 (lane 9, 10). Although both BKM120+TKI258 and BKM120+RAD001 exhibited very similar increases in cPARP1 levels, in VCaP cells, the level of S6 activation, only in the case of BKM120+TKI258, was similar to LNCaP cells treated similarly, Figure 2 (lanes 10, 11). The increased sensitivity of VCaP cells to BKM120+RAD001 combination is perhaps due to its intact PI3K-signaling when compared to LNCaP cells that have inactive PTEN resulting in upregulation of the pathway. The increase in S6 activation in VCaP cells upon BKM120+RAD001 treatment, when compared to LNCaP cells treated similarly needs further investigation. Taken together, however, our experiments suggest BKM120+TKI258 to be the combination treatment of choice to bring about the most synergy with complete PI3K pathway inhibition.

**Figure 7:**
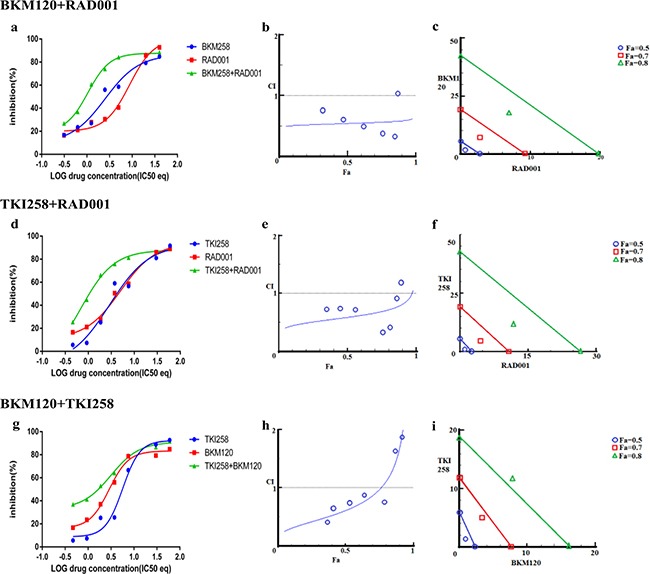
Combination effect of two drugs that target the PI3K-pathway simultaneously: PC3 cells were treated with the indicated combination of the drugs and analyzed as described in Methods and Figure 3

We next looked at the effect of drug-combination in CRPC lines. In the case of PC3 cells, treatment with BKM120+TKI258, BKM120+RAD001 or TKI258+RAD001 yielded CI value of 0.77, 0.65 and 0.54, respectively (Table 2 and Figure 7), suggesting high to moderate levels of synergy. However, unlike LNCaP cells, the CI-Fa plots in PC3 cells displayed a trend towards additive and beyond, especially at higher Fa values, Figure 7 (middle column). This was particularly prominent for the BKM120+TKI258 combination (Figure 7h) and was reflected in the highest CI (Fa 0.8) value of 1.14 (Table 2). To test if PC3 cells treated with the combination of BKM120+TKI258 would result in the greatest increase in cPARP1 levels, as previously observed in LNCaP and VCaP cells, we carried out western blot analysis upon treatment with the various drug combinations. As seen in Figure 4 (lanes 5 and 6) for PC3 cells, the most prominent cPARP1 bands observed were from cells treated with combinations of BKM120+TKI258 or BKM120+RAD001. To test if this was a consistent trend, we treated two other CRPC cells, 22Rv1 and Du145, both of which do not respond to AR antagonists. Consistent with results from LNCaP and VCaP cells, the BKM120+TKI258 combination elicited the highest amount of PARP cleavage (Figure 4, 22Rv1 lane 9, and Du145 lane 5). Taken together, our CI computation and biochemical data suggests that in prostate cancer a combination of BKM120+TKI258 is perhaps the most effective treatment modality to inhibit the PI3K-pathway resulting in maximum apoptotic response in the tested conditions.

Drug-antagonism can impair the efficacy of combination therapy. To further understand the trend towards antagonism observed in the case of BKM120+TKI258 combination (Figure 7h) we carried out a detailed study with increasing drug-concentration ratios in PC3 cells, Table 4. Although synergy is evident across most drug-ratios (CI values 0.03-0.89), it was strongly synergistic at lower concentrations of BKM120 and TKI258 (green boxes in Table 4). As the concentration of each drug increased, the synergy of combined treatment began to decrease with CI values ≥1 (yellow) and, at even higher drug concentrations, the effect shifted predominantly to antagonism (red). Perhaps the drug-target complexes are all completely saturated and thus become a limiting factor, or perhaps the drugs are somehow interacting with each other. Another possibility is that these drugs are no longer completely soluble at such high concentrations, leaving only a fraction of the drug available to inhibit the intended target. Nevertheless, this detailed analysis suggests that the effect of drug combination changes from synergy to antagonism when drug concentrations are increased. Thus, a careful analysis is imperative to designing a combination therapy to yield better response with reduced toxicity.

**Table 4:** Dose response matrix of BKM120+TKI2598 combination in PC3 cells

	BKM120									
	Conc (μM)		50	25	12.5	6.25	3.12	1.56	0.78	0.39
TKI258		Viability (%)	1%	8%	36%	39%	42%	64%	78%	84%
	50	4%	3.92	2.57	1.89	1.55	1.39	1.30	1.25	1.23
	25	15%	2.62	1.61	1.14	0.88	0.76	0.71	0.69	0.65
	12.5	26%	2.38	1.42	0.96	0.67	0.56	0.48	0.47	0.43
	6.25	51%	2.29	1.33	0.75	0.49	0.34	0.28	0.24	0.24
	3.12	67%	2.04	1.08	0.60	0.35	0.23	0.17	0.13	0.13
	1.56	76%	1.69	0.89	0.48	0.26	0.16	0.13	0.12	0.12
	0.781	83%	1.54	0.78	0.39	0.21	0.14	0.11	0.09	0.26
	0.39	93%	1.48	0.76	0.39	0.23	0.13	0.12	0.15	0.03

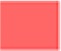
 Antagonism (CI > 1, red)

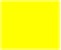
 Additive (CI = 1, yellow)

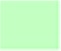
 Synergism (CI < 1, green)

### PC3 cells treated with MDV3100 and BKM120 or RAD001

Due to the recurrent loss of PTEN in PCa, BKM120, the inhibitor of PI3K, presents one of the most promising alternatives for PCa treatments. As mentioned above, the CI value can change with Fa and might result in reduced synergy between drugs, especially at high concentrations. To test whether an antagonistic effect existed between MDV3100 and BKM120 (as observed with BKM120 and TKI258 in PC3 cell) or MDV3100 and RAD001, we studied the two-drug combinations in PC3 cells, which have lost both AR and PTEN expression. The curve-shift plot in Figure 5a shows that combining BKM120 with MDV3100 is similar to the effect of BKM120 treatment alone. Similarly, the effect of combining MDV3100+RAD001 is the same as RAD001 alone, albeit slightly lower than BKM120 combination, Figure 5b. Thus, the presence of MDV3100 does not seem to affect the inhibitory property of BKM120 or RAD001.

### Effect of combining targeted therapy with chemotherapy

**Figure 8:**
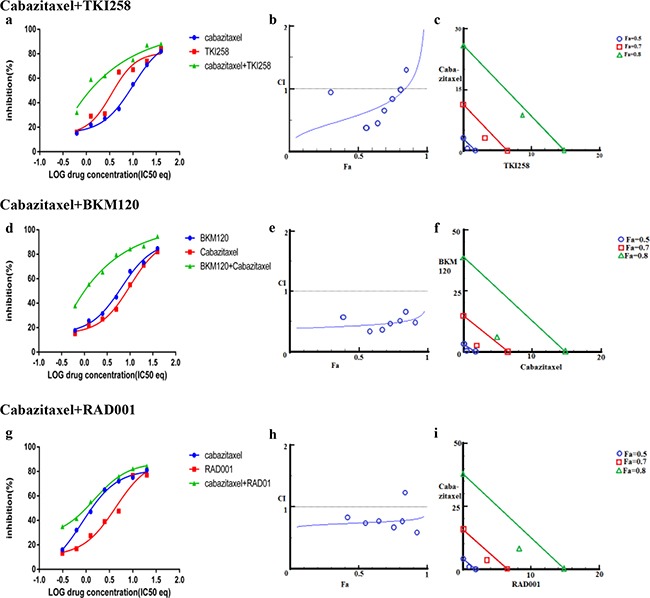
Combination effect of cabazitaxel with drugs that target the PI3K-pathway simultaneously: LNCaP cells were treated with the indicated combination of the drugs and analyzed as described in Methods and Figure 3

**Figure 9:**
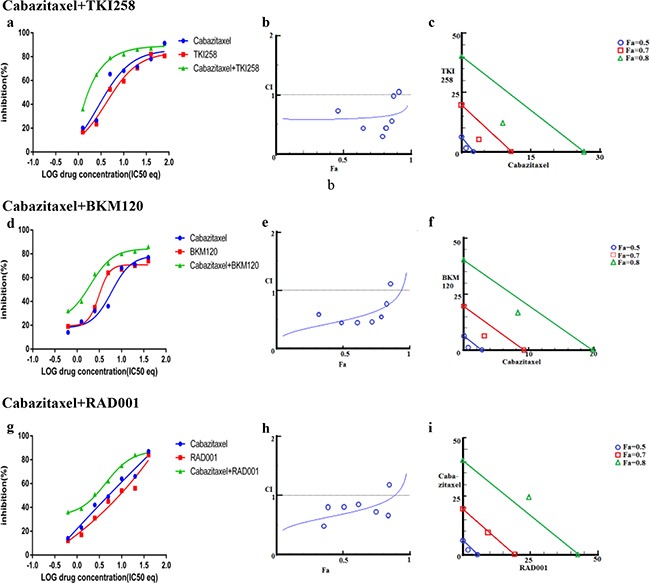
Combination effect of cabazitaxel with drugs that target the PI3K-pathway simultaneously: PC3 cells were treated with the indicated combination of the drugs and analyzed as described in Methods and Figure 3

In addition to testing the combination effect of targeted therapies, we investigated the combination effect of chemotherapy drug cabazitaxel with a PI3K-pathway inhibitor (BKM120, TKI258 or RAD001). Cabazitaxel is FDA approved drug to treat metastatic hormone refractory prostate cancer [4, 34]. When LNCaP and PC3 cells were treated with a PI3K-pathway inhibitor in combination with Cabazitaxel, comparable levels of synergism were observed in both the cell lines. The CI values ranged between 0.46-0.74 suggesting moderate to slight synergism (Table 2). In general, the CI value of cabazitaxel in combination with a targeted therapy was higher (less synergistic) than the CI value of two targeted therapies that included one of the same drugs, Table 2 and Figure 8 and 9(a, d, g). Interestingly, however, the cabazitaxel+BKM120 combination in LNCaP cells displayed no trends towards antagonism in the Fa-CI plot and distinct curve shift in the dose-response plot, Figure 8e. Intriguingly, in PC3 cells, the combination of cabazitaxel with BKM120 was more synergistic (CI 0.47) than BKM120+TKI258 (CI 0.77), Figure 8 and Table 2. Taken together, this suggests that using the combination of a PI3K inhibitor, especially BKM120, with cabazitaxel could have treatment benefit beyond that of using cabazitaxel alone. Overall, no significant antagonism was observed with any drug-combination in the PCa cell lines tested.

## DISCUSSION

A complex compensatory crosstalk between the AR- and PI3K-pathways has been identified, limiting the success of single-agent therapy targeting the individual pathways [23, 24, 35]. Indeed clinical trial of single agent RAD001 in chemotherapy-naïve patients showed only a moderate response [21], and all patients on MDV3100 develop disease resistance within 2 years [20, 36]. Thus, a combined treatment with inhibitors targeting the two pathways could act as a viable alternative [27]. Presently a clinical of combined inhibition of AR- and PI3K pathways, such MDV3100 and RAD001 is underway [37]. Other drugs (such as docetaxel, bicalutamide and ARN509) in combination with RAD001 are also in various phases of early clinical trial [37, 38].

Our data suggests that in AR positive conditions (LNCaP and VCaP cells) the BKM120+MDV3100 combination has the most synergistic effect with the ability to induce apoptosis. However, early clinical trail results with BKM120 (inhibitor of PI3K), either alone or with MDV3100, have not been very encouraging [22]. This could because the patients on the trial had already progressed on MDV3100. In this regards, our data suggests that combining BKM120+MDV3100 as first line therapy would be more efficacious than MDV3100 alone. The failure of single-agent PI3K pathway inhibitors suggests the complex and branched nature of the pathway requires better two-stage targeting to completely inhibit it. The focus remains at PI3K, which mediates the activation of AKT, along with mTOR a major effector of the TORC1 complex downstream of AKT. In this regard, our data also suggests that a BKM120+TKI258 combination to be more effective than BKM120+RAD001, and thus could have beneficial effect. Although there is an ongoing trail of BEZ235 (a dual PI3K - mTOR inhibitor) in combination with BKM120 [39], no clinical trial of BKM120+TKI258 in prostate cancer is being carried out.

### Antagonism and development of resistance

Although, at Fa 0.5 (50% cell death), we saw synergy in all the different combinations, we observed trends towards antagonism at higher Fa values especially in the case of BKM120+TKI258. For better therapeutic effect a threshold at Fa=0.8 (80% cell death) is most commonly used when investigating oncogenic drugs [40]. Interestingly, in microbial systems it has been shown paradoxically that antagonistic drug combinations lead to a reduction in the evolution of resistance [28, 41–43]. However, such drug-combination studies and their correlation with resistance-development have not been explored in cancer. This is of particular interest for PCa as it is a slow-progressing disease with a median age of diagnosis of 65 years. Keeping the tumor burden low or stagnant, with reduced chance of resistance development, than quick elimination of tumor load with increased chance of resistance development and compromised quality of life, could be a viable alternative.

Finally, we found synergistic effect of cabazitaxel in combination with any of the PI3K-pathway inhibitors, most prominently with BKM120 (Table 2). Furthermore, better synergy was observed when MDV3100 was combined with a PI3K-pathway, than when cabazitaxel was combined with the same PI3K-pathway inhibitor. However, similar synergies were observed when two-drugs targeting the PI3K-pathway were used or when a PI3K-pathway inhibiting drug was used in combination with cabazitaxel (Table 2). Taken together our study suggests that addition of PI3K/AKT/mTOR-pathway inhibition especially at the level of PI3K (with drugs such as BKM120) to existing therapies can enhance therapeutic efficacy. The study described here can be used in conjunction with newer techniques of culturing human tumor cells, such as in the form of organoids, to accurately identify best synergistic drug-combinations with potent efficacy and minimal side effects.

## MATERIALS AND METHODS

### Cell lines and reagents

Human prostate cancer cell lines (LNCaP, VCaP, CWR22Rv1 (referred to as 22Rv1), PC3, and Du145) were purchased from American Type Culture Collection (Manassas, VA, USA). RPMI 1640 media, phenol red-free RPMI 1640 media, Fetal Bovine Serum (FBS), Charcoal Stripped FBS One Shot, 1X phosphate buffered saline (PBS) and 100X Antibiotic-Antimycotic (A/A) solutions were purchased from Gibco-Invitrogen Corporation (Carlsbad, CA). Quick Start Bradford 1X dye reagent, 4X Laemmli sample buffer, a Mini Trans-Blot wet transfer system PVDF membranes and non-fat dry milk blocking grade blocker were purchased from Bio-Rad Laboratories (Hercules, CA). cOmplete, EDTA-free Protease Inhibitor Cocktail Tablets were purchased from Roche Diagnostics (Indianapolis, IN). PARP (9542), pAKT (2920), AKT (4691), pS6 (4858) S6 (2317) primary antibodies and secondary anti-mouse IgG (7076) and anti-rabbit (7074) horseradish peroxidase (HRP)-conjugated antibodies were purchased from Cell Signaling Technology, Inc. (Danvers, Massachusetts). Western Bright ECL western blotting detection kit was purchased from Advansta (Menlo Park, CA).

### Cell viability assay

Cell viability was assessed using the water-soluble tetrazolium salt (WST-1) assay (Clontech) following manufacturer's instructions. 5,000-10,000 cells/well were plated in a 96-well plate and after the cells had adhered the culture media was replaced with drug/s-containing media and cells further incubated for 72 hours. Thereupon, cell viability was assessed using the WST-1 protocol. The cell viability data was used to generate IC_50_ values using Prism 6 (GraphPad Software Inc, San Diego, USA). Experiments were always set in triplicates and repeated three times.

### Drug formulations and treatment

Buparlisib (BKM120, cat. S2247), Dovitinib (TKI-258, cat. S2269), Everolimus (RAD001, cat. S1120), Enzalutamide (MDV3100, cat. S1250) and Cabazitaxel (cat. S3022) were purchased from Selleck Chemicals (Houston, TX, US). All drugs were diluted in dimethyl sulfoxide (DMSO) (Sigma Aldrich, St Louis, MO) and aliquots stored in −80°C. Experiments were designed such that none of the inhibitors or inhibitor combinations would completely kill all the cells in any given experiments. The half maximal inhibitory concentration (IC_50_) values of each drug was determined by treating cells with a range of 0.012 – 200 μM concentration for three days. For combination studies, 0, 1/8, 1/4, 1/2, 1, 2, and 4 times IC_50_ of each drug and its combination was used in an array format. For BKM120-TKI258 studies in PC3 cells, both the drug concentrations ranged from 0–50μM in two-fold increments.

### Combination effect analysis

The fraction of viable cells remaining after three days of drug treatment was normalized to DMSO treated control and plotted against drug concentrations in the logarithmic scale. IC_50_ values were calculated by performing nonlinear regression analysis using Prism6. Although experiments were set in triplicates and replicated three times, only one set of representative data with the greatest best-fit curve was used for the computation.

### Combination index

The unified theory of using both linear and non-linear regression analysis, introduced by Chou, was used to evaluate the synergism, additivity and antagonism of the combination drug treatment [40]. Combination index (CI) values were calculated using the CompuSyn software (CompuSyn Inc., Paramus, NJ)[44] which uses the equation:

$$\text{Cl}=\frac{\text{CA},\text{x}}{\text{lCx},\text{A}}+\frac{\text{CB},\text{x}}{\text{lCx},\text{B}}$$

Where CA,x and CB,x are the concentrations of drug A and drug B in the combination to produce a certain effect X. ICx,A and ICx,B are the concentrations of drug A and drug B used as a single agent to produce that same effect.

### Interpreting drug-combination plots

CompuSyn also generates a plot of CI values at different fraction affected (Fa) levels referred to as Fa-CI plot or the Chou-Talalay plot, which are widely used to interpret drug combination effects [40, 45] and Supplementary Figure S1. A CI value of <0.1 indicates very strong synergism, 0.1–0.3 strong synergism, 0.3–0.7 synergism, 0.7–0.9 moderate to slight synergism, 1 nearly additive, 1.1–1.45 slight to moderate antagonism, 1.45–3.3 antagonism, and >3.3 strong to very strong antagonism. The relation of CI and Fa is depicted in Supplementary Figure S1A.

To further study the dose-dependent interaction of two drugs' isobolograms, particularly at Fa levels of 50%, 70%, and 80% inhibition were created, Supplementary Figure S1B. Since the single agents Fa value corresponds to IC_50_ value, the 50% isobologram of the combination provided direct comparison with single agent treatment. The 70% and 80% isobologram represent combination at a high effect level and have practical implications in therapeutic oncology [46]. Data points above or below the line of additivity indicate antagonism or synergism, respectively. Finally, to study the nature of inhibition with the drug combinations, a drug-effect-shift analysis of single-agent and combination was carried out, (Supplementary Figure S1C) which allowed for a direct comparison in terms of IC_50_ equivalents. Here synergy refers to a lowering of IC_50_ equivalent (left-shift) when compared to single agent curves on the same plot [47], and Supplementary Figure S1C.

### Western blot analysis

Prostate cancer cell lines (LNCaP, VCaP, 22Rv1, PC3, and Du145) were seeded in 12-well tissue culture plates and grown in complete RPMI 1640 media supplemented with 10% fetal bovine (FBS) and 1% antibiotic-antimycotic solution (A/A). When cells reached 80% confluence they were treated with 2xIC_50_ concentrations of MDV, BKM120, TKI258 or RAD001 alone or in combination for 24 hours. After treatment, excess media was removed and cells were collected and washed with 1X PBS after dissociation with 0.25% trypsin. For serum-starved experiments, LNCaP cells were first allowed to attach and grow in complete media until they reached 80% confluence. Cells were then cultured in phenol red-free RPMI 1640 media supplemented with 5% charcoal-stripped FBS and 1% A/A solution for 72 hours prior to treatment with drugs. All cells were maintained at 37°C in a humidified atmosphere containing 5% CO_2_ (Heracell™ 150i incubator).

Cell pellets were resuspended in RIPA buffer (50mM Tris,150 mM NaCl, 0.5% sodium deoxycholate, 1% NP-40) containing 1X protease inhibitor cocktail, and lysed by passing them five times through a 28G 1/2 needle. After lysis, the homogenates were centrifuged at 4°C for 20 min at 12,000 rpm and the supernatant collected. The protein concentration of the samples was determined by Bradford assay. Equal amounts of protein-lysate (35μg) were separated by SDS–PAGE electrophoresis on 8% gels for 2 hours at 100V. Following this, separated proteins were electro-transferred at 20V to PVDF membranes overnight (4°C) using a Mini Trans-Blot wet transfer system. The following day membranes were blocked in 1X TBST (20mM Tris-HCl pH 7.6, 150 mM NaCl, 0.1% Tween 20) containing 5% non-fat milk for 1 hour at room temperature. After blocking, membranes were incubated with primary antibodies as per manufacturer recommendations overnight at 4°C, followed by washing with 1X TBST (3 times x 5 minutes each) and probing with secondary HRP-conjugated anti-mouse or anti-rabbit antibodies (1:5000 dilution) for 1 hour at room temperature. After a final step of washing, proteins were visualized using Western Bright ECL detection kit reagents under a digital scanner Image Quant LAS 4000 by GE.

## SUPPLEMENTARY FIGURES AND TABLE




