# Supplementary material for: Combination effect of therapies targeting the PI3K- and AR-signaling pathways in prostate cancer
Source: Oncotarget. 2016 Oct 20;7(46):76181–96. doi: 10.18632/oncotarget.12771 (PMC5342806; doi:10.18632/oncotarget.12771)
Supplement: Supplementary file 1 [file oncotarget-07-76181-s001.pdf]

Combination effect of therapies targeting the PI3K- and AR-signaling pathways in prostate cancer

SUPPLEMENTARY FIGURES AND TABLE

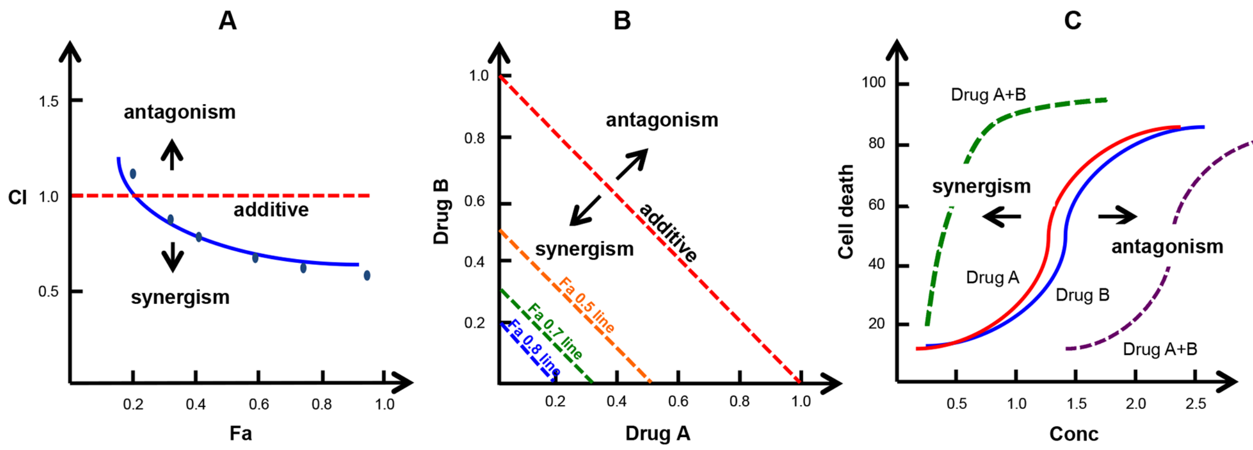

Supplementary Figure S1: Interpreting combinatorial plots. A. The Fa-CI plot: CI values were generated by non-linear regression methods and plotted against Fa (fraction affected/%inhibition). The horizontal red line indicates additive area. Above it indicates antagonism and values below synergism. Plot was generated using CompuSyn. **B.** Isobologram analysis. The diagonal, red-line indicates additivity, orange Fa 0.5 and blue Fa 0.8. Values that fall below these lines at the same Fa values (for example, 50% and 70% and 80%) suggest synergism, whereas when the fall above the corresponding line, it indicates antagonism. Plot was generated using CompuSyn. **C.** Combination dose-response curve: Values were normalized to IC50 values of the single agents (IC50 eq) using non-linear regression trendlines, and the data plotted. When dose-response curves of the combination (green line) shifted to left, it indicates synergism, whereas when it shifts to the right it suggests antagonism. Plot was generated using Prism 6.

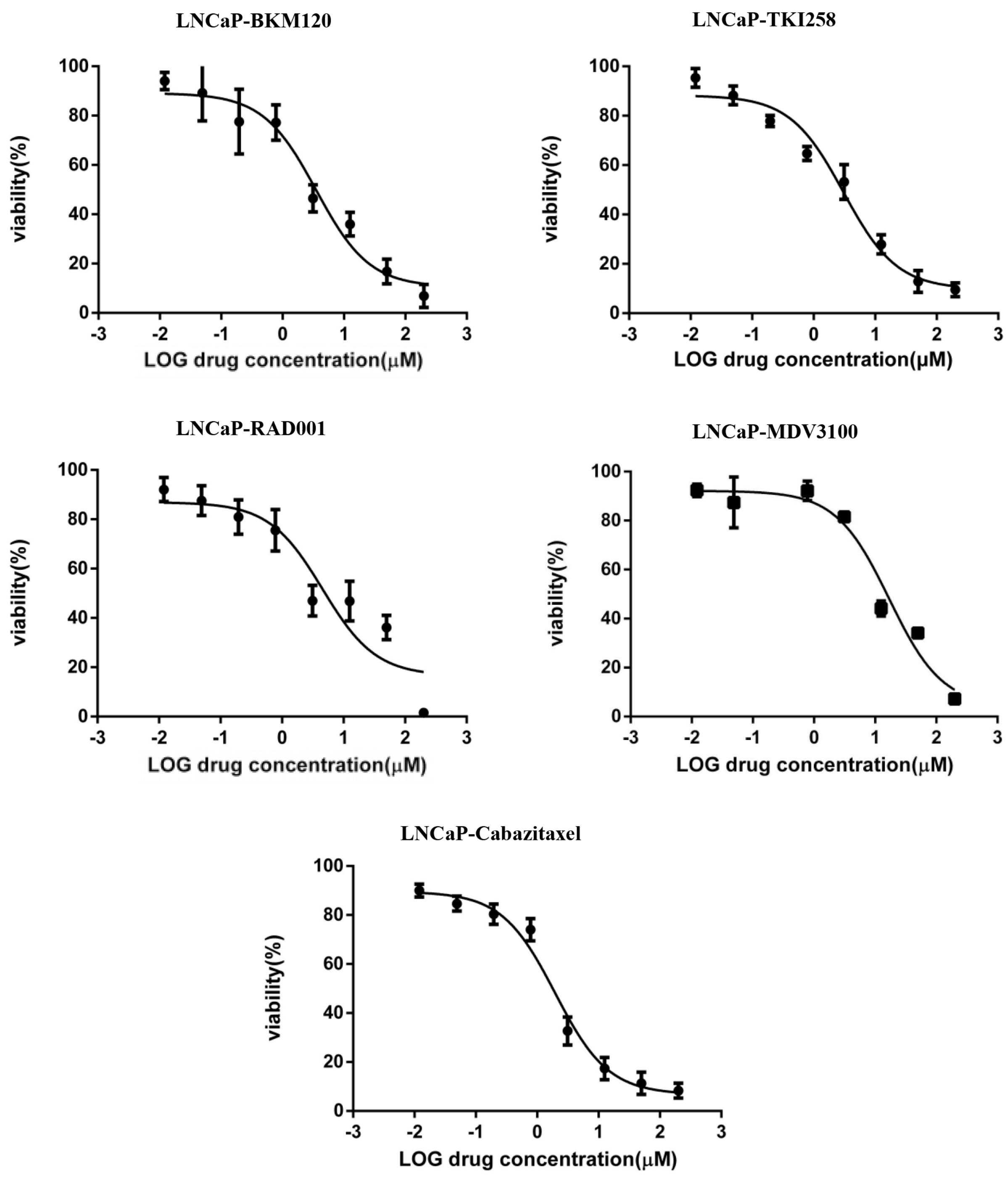

Supplementary Figure S2: Determination of single agent IC_{50} in LNCaP cells. Cells were treated with indicated drugs for 3 days and the number of surviving cells estimated using the WST1 assay. The dose-response values were then analyzes through Prism 6, using the non-linear curve fitting method. IC_{50} are presented in Table 1. Data points represent mean ±sd.

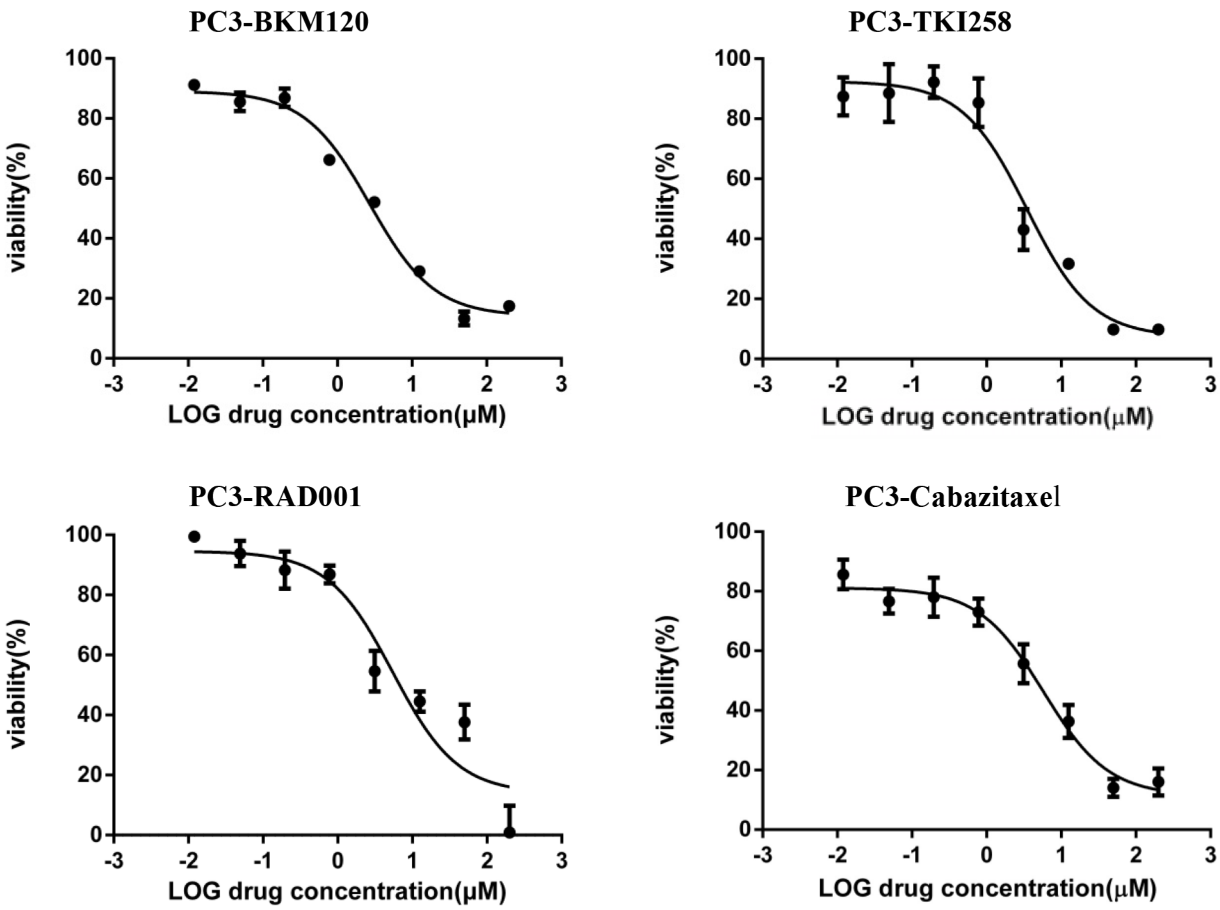

Supplementary Figure S3: Determination of single agent IC_{50} in PC3 cells. Cells were treated with indicated drugs for 3 days and the number of surviving cells estimated using the WST1 assay. The dose-response values were then analyzes through Prism 6, using the non-linear curve fitting method. IC_{50} are presented in Table 1. Data points represent mean ±sd.

22Rv1-BKM120

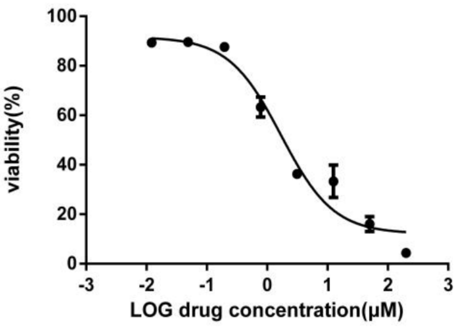

22Rv1-TKI258

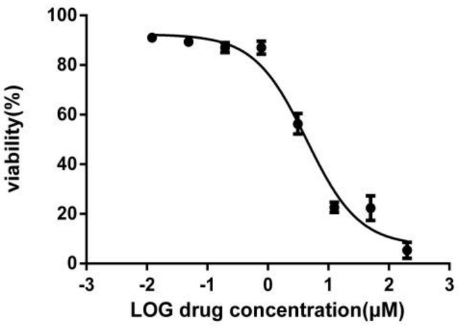

22Rv1-RAD001

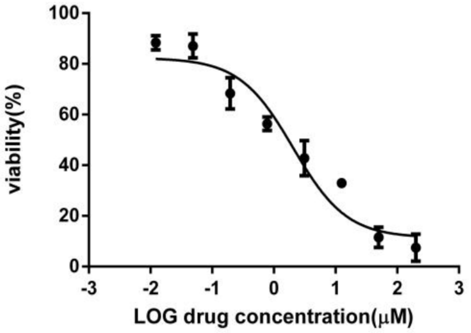

22Rv1-Cabazitaxel

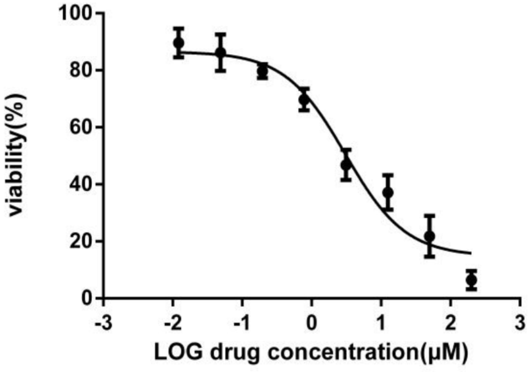

22Rv1-MDV3100

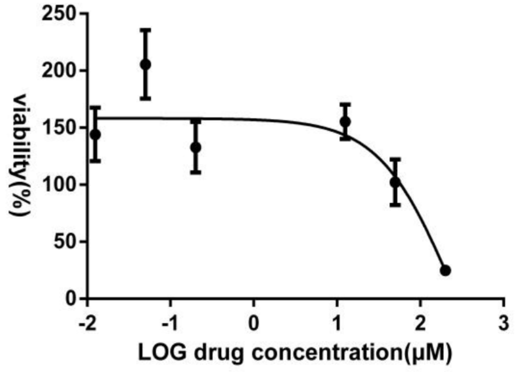

Supplementary Figure S4: Determination of single agent IC_{50} in CWR22Rv1 cells. Cells were treated with indicated drugs for 3 days and the number of surviving cells estimated using the WST1 assay. The dose-response values were then analyzes through Prism 6, using the non-linear curve fitting method. IC_{50} are presented in Table 1. Data points represent mean ±sd.

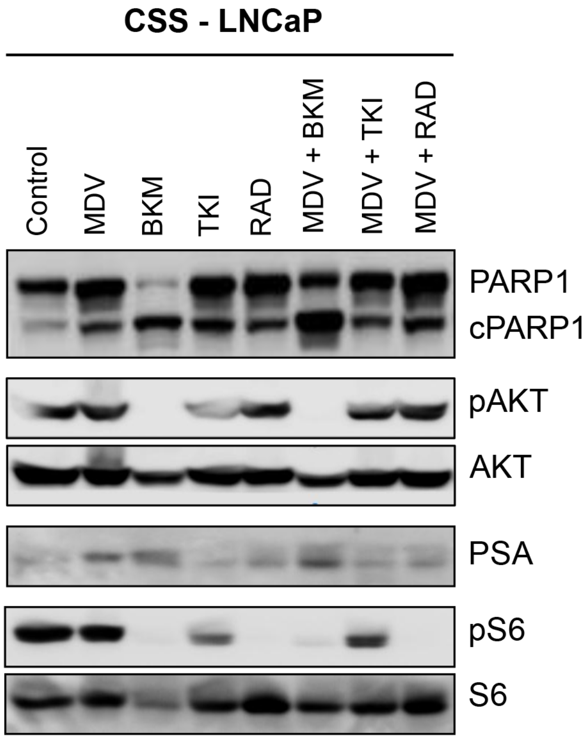

Supplementary Figure S5: Biochemical effect of perturbations with various drugs, affecting the AR- and PI3K-signaling pathways, and their 2-drug combinations in LNCaP cell growing in charcoal stripped serum. The cells were treated with IC50 drug concentrations of the mentioned drug(s) for 1 day and lysate of the treated cells was subjected to western blot analysis with the mentioned antibodies.

Supplementary Table S1: Single and combination IC50 with fold change estimations

See Supplementary File 1
