# Supplementary material for: Combination effect of therapies targeting the PI3K- and AR-signaling pathways in prostate cancer
Source: Oncotarget. 2016 Oct 20;7(46):76181–96. doi: 10.18632/oncotarget.12771 (PMC5342806; doi:10.18632/oncotarget.12771)
Supplement: Supplementary file 2 [file oncotarget-07-76181-s002.docx]

|  | | **MDV-TKI** | | **MDV-BKM** | | **MDV-RAD** | | **TKI-BKM** | | **TKI-RAD** | | **BKM-RAD** | | **Cab-BKM** | | **Cab-TKI** | | **Cab-RAD** | |
| --- | --- | --- | --- | --- | --- | --- | --- | --- | --- | --- | --- | --- | --- | --- | --- | --- | --- | --- | --- |
|  |  | **MDV** | **TKI2** | **MDV** | **BKM** | **MDV** | **RAD** | **TKI** | **BKM** | **TKI** | **RAD** | **BKM** | **RAD1** | **Cab** | **BKM** | **Cab** | **TKI** | **Cab** | **RAD** |
| **LNCaP** | Single | 6.31 | 3.04 | 6.31 | 3.23 | 6.31 | 4.05 | 3.04 | 3.23 | 3.04 | 4.05 | 3.23 | 4.05 | 1.80 | 3.23 | 1.80 | 3.04 | 1.80 | 4.05 |
|  | Combined | 0.15 | 0.46 | 0.11 | 0.35 | 0.07 | 0.21 | 0.32 | 0.32 | 0.33 | 0.33 | 0.60 | 0.60 | 0.89 | 0.45 | 0.35 | 0.70 | 0.64 | 1.29 |
|  | Fold reduction | 42.07 | 6.61 | 57.36 | 9.23 | 90.14 | 19.29 | 9.50 | 10.09 | 9.21 | 12.27 | 5.38 | 6.75 | 2.02 | 7.18 | 5.14 | 4.34 | 2.81 | 3.14 |
| **PC3** | Single |  |  |  |  |  |  | 2.58 | 2.82 | 2.58 | 5.46 | 2.82 | 5.46 | 6.32 | 2.82 | 6.32 | 2.58 | 6.32 | 5.46 |
|  | Combined |  |  |  |  |  |  | 1.24 | 0.82 | 1.73 | 0.86 | 1.64 | 0.65 | 1.41 | 0.71 | 1.31 | 0.99 | 2.01 | 2.01 |
|  | Fold reduction |  |  |  |  |  |  | 2.08 | 3.44 | 1.49 | 6.35 | 1.72 | 8.40 | 4.48 | 3.97 | 4.82 | 2.61 | 3.14 | 2.72 |
| **22RV1** | Single |  |  |  |  |  |  | 0.97 | 0.23 | 2.83 | 1.02 | 0.06 | 0.23 |  |  |  |  |  |  |
|  | Combined |  |  |  |  |  |  | 0.09 | 0.15 | 0.57 | 0.2 | 0.01 | 0.04 |  |  |  |  |  |  |
|  | Fold reduction |  |  |  |  |  |  | 10.78 | 1.53 | 4.96 | 5.1 | 6.00 | 5.75 |  |  |  |  |  |  |

Supplementary Table S1: Single and combination IC50 with fold change estimations
